# Sugar Tax or What? The Perspective and Preferences of Consumers

**DOI:** 10.3390/ijerph191912536

**Published:** 2022-10-01

**Authors:** Agnieszka Piekara

**Affiliations:** Department of Bioprocess Engineering, Wroclaw University of Economics and Business, Komandorska 118-120, 53-345 Wrocław, Poland; agnieszka.piekara@ue.wroc.pl; Tel.: +48-713680872

**Keywords:** sugar tax, health promotion, attitudes, obesity, food economy

## Abstract

Reducing high-calorie food and beverages consumption is a multi-dimensional challenge spanning agriculture to food marketing. Adverse health effects resulting from sugar-sweetened beverages such as obesity, diabetes, cardiovascular disease or dental carries have been described on numerous occasions. Poland is one of the countries that have introduced the sugar tax. The study aims to understand the degree of consumers’ awareness of the upcoming changes and their opinions and assessments of the efficiency of various activities. The study was based on Computer-Assisted Web Interview (CAWI). The sample comprised 500 adult consumers. Most of the respondents (69.6%) are aware that a new charge for sweetened beverages is going to be introduced, and for 78.9% of the respondents, it is important to take action aimed at reducing the consumption of sweetened beverages by consumers. Well-educated respondents as well as women perceive a greater degree of need to take specific action within the area of health policy (*p*-value 0.010 and 0.000 respectively). The sugar tax is considered an effective tool for limiting the purchase of sweetened products. Other types of activities within the framework of preventative healthcare that aim to reduce the consumption of sugar by society should also be developed.

## 1. Introduction

Reducing high-calorie food and beverages consumption is a multi-dimensional challenge spanning agriculture to food marketing. Sugar consumption varies from country to country (nationality). It also depends on several sociodemographic factors [1]. The adverse health effects related to sugar consumption (including consumption of sugary beverages) such as obesity, nonalcoholic fatty liver disease, diabetes, cardiovascular disease, or dental carries were described in numerous publications [2,3,4,5]. In the last several years, sugar consumption has been a subject of public health-related debates aimed at answering the following question: how to efficiently decrease the intake of added/free sugar in society. Across the world, many strategies have been used, including sugar tax [6], nutritional claims, warning labels [7,8], or imposing limits on the TV and radio advertising of high-sugar products, especially prohibiting the marketing of high-sugar products to children [9,10]. Quite often, individual governments choose fiscal policy tools such as sugar tax as the first action aiming to decrease, e.g., purchasing sugar-sweetened beverages, which has also been recently recommended by the WHO. More than 25 countries have introduced a sugary drinks tax, including at least 11 in Europe [11]. Poland is among these countries. Since January 2021, based on “The Act on the Amendment of Certain Acts in Connection with Promotion of Healthy Consumer Choices“, Poles are required to pay a tax on soft drinks that contain sugar. The new tax comprises a fixed fee for the content of sugar, which equals or is lower than 5 g in 100 mL of a beverage, or for the content of at least one sweetening agent, mentioned in Regulation No 333/2008 in any quantity. It also includes a variable fee for every gram of sugar over 5 g in a 100 mL beverage. Both The European Food Safety Authority (EFSA) and the US Institute of Medicine claim that they are unable to precisely set an upper limit for the intake of sugar [12,13]. However, many health-related organizations, including the organizations mentioned above, suggest reducing the intake of free sugars. The WHO has recommended reducing the intake of free sugars to <10% of total energy (TE), and the Scientific Advisory Committee on Nutrition UK (SACN) has suggested a further reduction in free sugars to below 5% of total energy intake [14,15]. The food industry uses a wide variety of calorific sweetening agents (CSA) and low or non-caloric agents to obtain desirable organoleptic properties [16]. It can be a challenge for consumers to successfully identify all of them when they must combat the misleading marketing information concerning the amount of sugar that is added to the products [17,18]. It is the first research of such type in the context of sugar tax conducted on the Polish market. Dynamic policy changes require the observation and study of consumer behaviors.

In countries where sugar tax had already been introduced, consumers showed either a positive attitude (they supported it) or a negative attitude (they denied the sense and legitimacy of the tax) [11,19,20]. Additionally, it was reported that consumers were unaware of the new tax [21]. In situations where the revenue was used to introduce improvements to the healthcare system, society was more supportive of the new tax model [20,22]. According to the declaration of the Polish government, financial resources obtained from the sugar tax will be used to finance the provision of healthcare, prevention, education, as well as other programs which aim to fight against diseases, especially lifestyle diseases such as overweight and obesity [23]. It was shown that actions other than fiscal changes, such as calorie labeling or removing drinks from schools, could garner greater public acceptance [24]. The school-based limitations that were also introduced in Poland in 2015 consisted of restricting sales of sweetened beverages and other snacks in educational institutions [25]. In many cases the positive impact of the sugar tax cannot be ignored. Examples from Mexico, Chile, and Hungary showed that SSB purchasing levels decreased by smaller or higher values (or for a shorter or longer period of time); however, more action is needed to reduce obesity and noncommunicable diseases (NCDs) [26,27,28].

The aim of the study is to understand the respondents’ opinions on the activities aimed at reducing the quantity of sugar consumed by society. It is also aimed at understanding the degree of consumers’ awareness of the upcoming changes, and their opinions and assessments on the efficiency of various activities. 

The following hypotheses were formulated for the purpose of this study:

**Hypothesis** **(H1).** *The general public does not possess knowledge on the subject of the introduced sugar tax*;

**Hypothesis** **(H2).** *It is necessary to take action aimed at reducing the consumption of the sweetened beverages by the consumers*;

**Hypothesis** **(H3).** *The introduction of new regulations may change consumer behaviors (declaration)*;

**Hypothesis** **(H4).** *Consumers believe that other types of actions within the framework of preventative healthcare which aim at reducing the consumption of sugar by society would be more successful*.

## 2. Materials and Methods

The study was based on the CAWI, which stands for Computer-Assisted Web Interview. The sample comprised 500 adult Poles. This sampling quota was set to ensure that the respondents’ structure was reflective of the demographic variables for Polish society. An anonymous, self-administered online questionnaire was used to collect the data. The data were collected between May and June 2020 via an anonymous, self-administered online questionnaire that was developed by the author and based on a literature review, preliminary interviews with consumers and available market reports. The survey form was made available to respondents as a link that could only be used once to avoid one person completing the survey several times. Only completed forms were used for further analysis, and the results were also checked, among others, to ensure no results with only one type of answer. The questionnaire was divided into two parts. The first part consisted of sociodemographic questions; the second part included questions focused on the respondents’ opinions on the activities aimed at reducing the consumption of sugar, including their awareness of the upcoming changes. To reduce the risk of respondents struggling to understand the questions, validation was conducted. Pre-testing was conducted on a group of 10 consumers (of different ages, genders, education, and wealth levels) to reject the statements that were considered irrelevant. The reliability of the test tool was conducted by the Kaiser–Meyer–Olkin test and the Barlett’s test, and the results were 0.829 and *p* < 0.0001, respectively.

The questionnaire was addressed to adult Poles (older than 18). The only criteria were the amounts provided in the sample distribution. The study was continued until reaching the sample quota (*n* = 500) (Table A1). The invitation was initially sent out to 73,217 potential responders. Four questionnaires were rejected due to not being filled out completely. The relationships were confirmed using the chi-squared test (*p* ≤ 0.05) and the proportions test. The proportions test is used to compare two variables. By comparing two answers to a given question, one can learn which of the responses is chosen significantly more frequently than the other. The chi-square independence test was used to confirm the statistical significance between the compared variables. Cronbach’s alpha coefficient for the attitude questions was 0.845. The results of the survey were subjected to a statistical analysis using PQStat (version 1.6.4, PQStat Software, Poznań, Poland) and Statistica (version 12, StatSoft Poland, Krakow, Poland).

## 3. Results

The proportions test was used to verify the H1 hypothesis. The obtained results demonstrate that the vast majority of the respondents (69.6%) are aware that a new charge for sweetened beverages will be introduced (Table A2). The proposed hypothesis (H1) was rejected based on this conclusion. The chi-square independence test (*p*-value 0.002) proved the statistical significance between the gender of the respondents and their knowledge about the introduced sugar tax. In Poland, men are more aware of the policy changes (75.8%) than women (62.9%). The age structure of the respondents was taken into consideration when checking the sources of information on the planned policy changes (regarding sweetened beverages).

**Figure A1:**
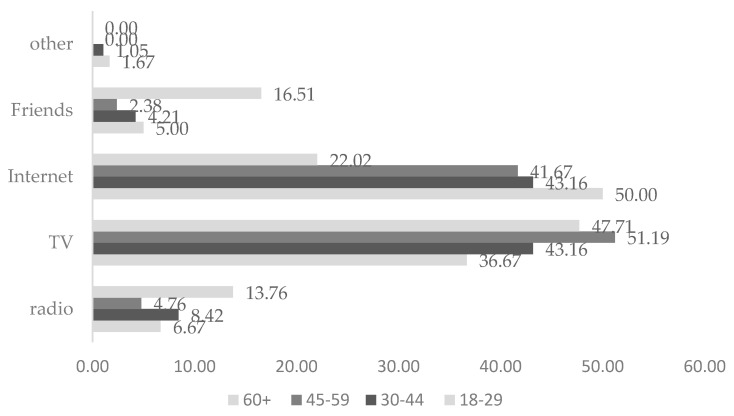
Sources of respondents’ knowledge of sugar tax, taking into consideration the age structure [%] *n* = 500.

The majority of Poles found out about the planned changes from mass media such as radio, TV, and the Internet (91.7%). The Internet was the predominant source of information on the subject for the youngest respondents (50.0%). The respondents aged 45–59 and the respondents from the eldest age group named TV as their source of information on the subject of the sugar tax (51.2% and 47.7%, respectively) (Figure A1). The respondents note that it is necessary to take action that aims to reduce the consumption of sweetened beverages by consumers (52% of respondents), while almost half as many respondents (27.8%) acknowledge that this is necessary, but only for children (Table 1). Only, or as many as 20.02%, of all respondents have no opinion or do not agree with the health policy. This should be taken into consideration in discussions on the subject, since many people do not perceive it to be a problem. The second hypothesis (H2) was confirmed and verified (Table A3).

The chi-square independence test (*p*-value 0.010) confirmed that the relationship between ‘perceiving the necessity to take action intended at reducing consumption of sweetened beverages’ and ‘the level of education of the respondents’ has statistical significance. The analysis of the answers led to the conclusion that Poles with a higher education (63.1%) perceive the need to take specific action within the area of health policy to a greater degree than people with an education level lower than secondary (47.4%), or secondary (47.2%). The chi-square independence test (*p*-value 0.000) proved the statistical significance between the gender of the respondents and their opinion about the need to reduce the SSB consumption.

Next, the respondents were asked: “Introducing a new charge is a part of the health-promoting policy. Do you believe it can be an efficient way of reducing the consumption of sweetened beverages by the consumers?” (Table 2). The majority of Poles (51.0%) claim that the additional charge on sweetened beverages will be an efficient way of reducing their consumption (Table 3). The chi-square independence test (*p*-value 0.003) proved the statistical significance between the gender of the respondents and their opinion about the effectiveness of the sugar tax (Table 3).

As many as 34.6% of consumers (*n* = 500) declared that they will not limit buying SSB, and 29.2% are not sure how the sugar tax will affect their behavior. Such research should be repeated after the sugar tax is introduced.

Another hypothesis formulated during the study stated that the introduction of new regulations may change consumer behaviors (H3) (Table A4). Some consumers declared that they will check the content of sugar or other sweetening agents in the product of their choice. This hypothesis can be confirmed based on the statistical analysis and the results of the test of proportions. Generally, the majority of the respondents concluded that the additional charge will influence their behavior. No significant differences in the declaration of SSB label use were observed among all demographic characteristics examined in this study.

Other known types of actions are available within the framework of health policy. These actions are aimed at reducing the consumption of sugar and changing the dietary habits of the consumers. For this reason, the final part of the study focuses on understanding the opinions of respondents on the subject of these actions. The respondents were presented with eight attitudes (Table 3) on a 5-point scale, where five meant ‘completely agree’ and 1 ‘completely disagree’ (Cronbach’s Alpha Value 0.845).

The respondents found the following actions to be the most effective:

- Placing information about the contents of sugar on the packaging of the sweetened beverages in a big font—76.4% of responses;
- Using graphics to display information about the sugar contents on the packaging of the sweetened beverages—74.2% of the responses;
- Requiring information to be provided about the health effects of the excessive consumption of sugar when running advertisements—73.4% of the answers.

The introduction of an additional charge which increases the price of sweetened beverages was selected to be an effective action; -slightly more than half of the respondents agreed.

A hypothesis was formulated, which stated that consumers believe that other types of preventative healthcare actions that aim to reduce society’s consumption of sugar would be more successful (H4). The results of the test proved a statistical significance for five out of seven analyzed actions (Table 4).

## 4. Discussion

There are at least two methods of SSBs taxation: mean, based on the value of the purchase (ad valorem tax) introduced, e.g., in Chile [27]; or on the quantity purchased (in the case of SSBs, the volume of the sugar content in the drink [29]). The evidence of the efficacy of these interventions includes a reduction in purchases of SSBs (Mexico, Chile) [30] or an increase in sales of untaxed beverages and water (USA) [31]. Media coverage related to the introduction of the sugar tax in Poland is so extensive that most of the respondents (almost 70%) knew about the soon-to-be introduced changes in the law, which came into effect on 1 January 2021. The Internet was the primary source of information about the sugar tax for the young respondents. The older respondents mentioned television to be the primary source of information. It can be concluded that the media coverage was wide enough to reach the public. An opposite effect was observed by Altman et al., who found that the awareness of SSB taxes was low overall in an analyzed group of consumers from the USA [32]. The positive role of the Internet in shaping consumers’ food choices cannot be neglected. Another research result claims that the Internet can be crucial and has a positive influence on weight control [33].

Information regarding this subject has been published many times because the Polish government decided to postpone introducing the tax by several months due to the COVID-19 pandemic. The majority of the respondents believe introducing a new charge as a part of the health-promoting policy to be an effective and sufficient means of reducing the consumption of sweetened beverages by consumers. This view dominates among younger respondents and among people who declare having a good financial situation. A general decrease in the consumption of SSB was observed in countries that introduced the sugar tax. The observed results show the relationship between the introduction of the tax and a notable decrease in soda consumption and the consumption of energy drinks [34,35]. Polish authorities ensure that tax revenues will be spent on the promotion of healthy consumer choices. The same to increase public acceptance of the new tax was suggested by Eykelenboom et al. [22], whose research results show that in general, 40% of the Dutch population (*n* = 500) support SBB taxation (51% of Poles participated in this study). In contrast, according to the studies conducted by Bombak et al., inhabitants of Michigan (USA) suggested that a sugar-sweetened beverage tax would be unsuccessful and would not change the shopping habits of regular consumers of SSB [36].

Naturally, decreasing the consumption of beverages with high sugar content is one of the objectives of introducing the sugar tax. However, it is necessary to observe this from a global perspective to ensure that such action will also influence other aspects of consumers’ behaviors, which may have an even better effect as part of obesity prevention measure [37]. This is why the respondents were asked whether they would change their habits related to reading labels and checking the amount and type of sweetening agent used in the product. Following UE 1169/2011 Regulation, the entity introducing a food product must place information on the packaging concerning nutritional value, which also applies to SSB. Based on the obtained results, it can be concluded that the positive effect of the sugar tax introduction may increase the consumers’ awareness about the beverages they drink.. Over half of the respondents declare that after introducing the additional charge, they will check the content of sugars and other sweetening substances in beverages. Due to this, it will be possible to make better nutritional/food choices. In general, the results confirmed Bryła’s findings that women read food labels more often than men, and that the age of respondents does not corelate with label reading [38].

Despite many advantages of introducing the sugar tax, doubts continue to emerge. The majority of the respondents see the benefits of introducing the tax, but at the same time, they believe that other actions could be more effective as part of the health preventative care aimed at the consumption of sweetened beverages by society. One of the actions assessed as potentially effective was ‘the ban on advertisements of sweetened beverages during the hours when the highest number of children are teenagers are watching’. Children and teenagers are a group of consumers who are most exposed to dishonest marketing and do not have the knowledge and skills to look critically at the messaging they are subjected to. They are exposed to a high number of food commercials broadcasted on TV on an everyday basis, which can have a negative influence on children’s eating behaviors [9,39]. 

The results of the study (Table 3 and Table 4) are in line with interventions analyzed by Hagmann et al. [7], where health campaigns and nutritional information on the packaging received the highest level of public support [7]. Consumers from the UK and the USA also preferred education than taxation as a way to reduce SSB consumption [40]. The study participants showed an interest in the idea of including information about the health effects of sugar consumption (similar to the packaging of tobacco products)in commercials and on the packaging of SSB. Scaring the consumers with depictions of illnesses and deteriorating health may be a more straightforward and effective message, even though the respondents believe that providing information about the calorific value of the product may be even more effective than the sugar tax. It was proven that the respondents do not find the warning about the hi-sugar value to be effective [41] and understanding the typical nutritional information presented on food labels may be inadequate and hard to understand by consumers [42]. The packaging of tobacco products includes warnings stating that smoking can end in death and have other frightening effects. On the other hand, nutritional warnings only include information on the content of the product [43,44]. Despite the strategy chosen by a given government, it is important ensure the primacy of public health goals. The opposite direction to prohibition and limitations is the extensive promotion of healthy habits. As proven by Denver and Christensen, people with higher organic consumption consume less fat/confectionary products [45]. 

Another problem is the placement of the warning, which may be ineffective. The appropriate design and content of the warnings can increase their visibility and effectiveness [46,47]. A well-designed warning label can help to combat obesity especially, and some studies suggest that on a daily basis, consumers are subjected to many messages about low-nutrient foods—particularly, close to the point of sale [48]. On the other hand, it has been already proven that labels including health warnings—especially those that include a combination of image and text—are likely to decrease the number of energy-dense snacks purchased online [10]. The analysis of the answers provided by the group of Polish respondents allowed us to conclude that they see the need to take action aimed at reducing the consumption of sweetened beverages. 

According to the estimations, the decrease in sales of carbonated beverages between January and August 2021, in comparison to the same period in 2020, exceeded 20%. At the same time, the prices of such products increased by 36%. The prices of flavored water also increased by 33%, with a 21% decrease in sales volume. A different trend was observed when looking at energy drinks. An increase in the average price per liter of about 15% was reported. This increase was observed along with the 4% increase in sales compared to 2020. The introduced tax was not the only factor that influenced the reduction in demand for the selected SSB—the pandemic and the lower temperatures observed during this period were other meaningful factors [49]. Long-term market observations are needed to assess the importance of the sugar tax on Poles’ behavior. Many other factors should also be taken into consideration, such as economic situation, inflation, weather and so on. It is worth monitoring whether the sugar tax revenue changes according to the premises.

Limitations and future research: All of the data were self-reported, leading to the possibility that the respondents may have misreported some data. The setting was artificial and the study task hypothetical. Another limitation of the study is the limited sample size. Making more observations would enable a more precise analysis, especially in the context of income. It would be valuable to repeat this study to learn how the introduction of sugar tax changed the respondents’ behavior and opinions about it.

## 5. Conclusions

Most respondents are conscious of the changes concerning the introduction of an additional charge for sweetened beverages. The majority of Poles declare that they will limit purchasing sweetened beverages after the prices are raised (58.4%), and after introducing the additional charge, they will check the content of sugar and other sweetening agents (55.4%). According to the respondents, it is necessary to take health policy-related action that aims to reduce the consumption of sugar. The introduction of an additional charge is perceived to be effective by Poles. However, they have pointed to other actions as being more effective: placing information about the contents of sugar on the packaging of sweetened beverages using a big font (36.4%); requiring information to be provided about the health effects of the excessive consumption of sugar when advertising (33.4%); using graphics to display information about the contents of sugar on the packaging of the sweetened beverages; requiring information to be provided about the health effects of the excessive consumption of sugar when running advertisements (32.4%).

The sugar tax is considered an effective tool for limiting the purchase of sweetened products, thus, influencing a reduction in the amount of sugar in consumers’ diet. However, it is necessary to consider the long-term influence accompanying fiscal policy. Will consumers forget, in the next 5 to 10 years, that the price of a particular beverage increased as a result of imposing the so-called sugar tax? Is it possible that the young consumers who are yet to enter the consumer market will not be at all aware of the existence of such taxation? Another question for future research could be: does the long-term educational and awareness-raising activities, or the combination of the tax and mandatory warning labels and limitations imposed on marketing have a measurable effect?

## Figures and Tables

**Table 1:** Consumers’ opinion on the need to take action to reduce the consumption of sweetened beverages (H2 *n* = 500).

	Yes [%]	Yes, Only among Children [%]	No [%]	I Don’t Know [%]	*p*-Value *
General	52.0	27.8	14.0	6.2	
Gender					**<0.001**
Women	62.1	23.3	7.5	7.1	
Men	42.7	31.9	20.0	5.4	
Age					0.753
18 to 29	47.1	32.2	13.8	6.9	
30 to 44	49.0	25.5	18.1	7.4	
45 to 59	54.3	27.6	12.9	5.2	
Over 60	56.1	27.7	10.8	5.4	
Education					**0.010**
Lower than secondary	47.4	30.9	17.1	4.6	
Secondary	47.2	33.0	11.4	8.5	
Higher	63.1	18.1	13.4	5.4	

* chi-square test, *p*-value < 0.05. Bold fonts: Statistically significant difference.

**Table 2:** Distribution of the responses to the question: Introducing a new charge is a part of the health-promoting policy. Do you believe it can be an efficient way of reducing the consumption of sweetened beverages by the consumers?

	Yes [%]	No [%]	I Don’t Know [%]	*p*-Value *
General	51.00	40.2	8.8	
Gender				*p* = 0.003
Women	57.9	32.5	9.6	
Men	44.6	47.3	8.1	
Age				*p* = 0.389
18 to 29 years old	59.8	36.8	3.4	
30 to 44	50.3	38.9	10.7	
45 to 59 years old	50.9	39.7	9.5	
Over 60	46.6	43.9	9.5	
Education				*p* = 0.226
Lower than secondary	47.4	43.4	9.1	
Secondary	55.1	34.1	10.8	
Higher	50.3	43.6	6.0	

* chi-square test, *p*-value < 0.05.

**Table 3:** Assessment of the effectiveness of actions taken to reduce the consumption of sugar, *n* = 500.

Statement	5 * [%]	4 * [%]	3 [%]	2 [%]	1 [%]	Sum of 4 & 5 [%]	*p*-Value *
15.03 Placing information about the content of sweetened beverages on the packaging of sweetened beverages using a big font	36.4	40.0	10.4	10.6	2.6	76.4	0.025
15.08 The need to provide information about the health effects of sugar consumption in graphic form when running advertisements	33.4	40.0	14.0	9.4	3.2	73.4	0.022
15.04. Placing the information about the content of sugars using graphics on the packaging of the sweetened beverages	32.40	41.8	12.2	11.0	2.6	74.2	0.041
15.07 Requirement to provide information concerning the content of sugar in the product when running an advertisement	29.4	40.6	15.0	12.8	2.2	70.0	0.028
15.05. Placing information concerning the calorific value of the product on the packaging of the product using graphics	28.0	42.8	14.6	11.8	2.8	70.8	0.035
15.06 Placing information about the health effects of sugar consumption on the packaging of beverages	27.8	37.0	17.2	37.0	27.8	64.8	<0.001
15.02 Ban on advertisements of sweetened beverages during the hours when the highest number of children and teenagers are watching	19.8	33.4	16.4	20.8	9.6	52.2	<0.001
15.01 Introducing a new charge on sweetened beverages is an effective element of health policy	11.4	39.6	10.2	22.2	16.6	51.0	<0.001

* Five-point scale statements, where 1—‘strongly disagree’; 2—‘I rather disagree’; 3—‘I have no opinion’; 4—‘I rather agree’; 5—‘I definitely agree’. Percentage of responses 4 and 5; DSs—dietary supplements; Cronbach’s Alpha Value—0.845; * Statistically significant difference: *p* ≤ 0.05 (chi2 test).

**Table 4:** The results of the chi-square test used to verify the formulated hypothesis (H4 *n* = 500).

		1. Introducing a New Charge on Sweetened Beverages Is an Effective Element of Health Policy				*p*-Value *
		No		Yes		
		N	%	N	%	
2. Ban on advertisements of sweetened beverages during the hours when the highest number of children and teenagers are watching	No	91	50.8	57	26.6	**<0.001**
	Yes		49.2	157	73.4	
3. Placing information about the content of sweetened beverages on the packaging of sweetened beverages using a big font	No	35	19.0	28	12.0	**0.045**
	Yes	149	81.0	206	88.0	
4. Placing the information about the content of sugars using graphics on the packaging of the sweetened beverages	No	34	19.3	31	13.1	0.089
	Yes	142	80.7	205	86.9	
5. Placing information concerning the calorific value of the product on the packaging of the product using graphics	No	40	22.7	31	13.7	**0.018**
	Yes	136	77.3	196	86.3	
6. Placing information about the health effects of sugar consumption on the packaging of beverages	No	57	35.0	29	12.7	**<0.001**
	Yes	106	65.0	200	87.3	
7. Requirement to provide information concerning the content of sugar in the product when running an advertisement	No	36	21.4	33	14.3	0.065
	Yes	132	78.6	197	85.7	
8. The need to provide information about the health effects of sugar consumption in graphic form when running advertisements	No	33	18.9	26	11.4	**0.034**
	Yes	142	81.1	203	88.6	

* Statistically significant difference: *p* ≤ 0.05 (chi2 test). Bold fonts: Statistically significant difference.

## Data Availability

The data presented in this study are available on request from the corresponding author. The data are not publicly available due to ongoing investigations.

## Appendix A

**Table A1:** The respondents’ demographic information.

	N	%
Gender		
Women	240	48.0
Men	260	52.0
Age		
18 to 29	87	17.4
30 to 44	149	29.8
45 to 59	116	23.2
Over 60	148	29.6
Education		
Lower than secondary	175	
Secondary	176	35.2
Higher	149	28.8
Material situation		
Lives very well and can afford some luxury	12	2.40
Lives well and can afford a lot without saving	155	31.0
Lives on a moderate level, can afford everyday expenses but must save to purchase something bigger	293	58.6
Lives modestly, must save on an everyday basis	32	6.4
Lives very modestly and lacks money to satisfy basic needs	5	1.0
Hard to say	3	0.6

**Table A2:** The results of the proportions test conducted to verify the set hypothesis H1 *n* = 500. (Question: Have you heard about the ongoing works on the introduction of the so-called “sugar tax” in Poland, an additional fee on the price of sweetened beverages?).

Answer	[%]	Expected	*p*-Value *
yes	69.6%	51%	**<0.001**
no	30.4%	49%	**<0.001**

* proportion test, *n* = 500. Bold Fonts: Statistically significant difference.

**Table A3:** The results of the proportions test conducted to verify the set hypothesis H2 *n* = 500. (Question: Do you think that action should be taken to reduce the consumption of sweetened beverages by consumers?).

Answer	[%]	Expected	*p*-Value *
Yes **	79.8	51	**<0.001**
No	20.2	49	

* proportion test, *n* = 500; ** sum of answers: yes; yes, only among children. Bold Fonts: Statistically significant difference.

**Table A4:** The results of the proportions test used in order to verify the H3 hypothesis *n* = 461.

Are You Going to Check the Content of Sweetening Agents after Introducing the Additional Charge?	%	Expected	*p*-Value *
Yes	60.09	51%	**<0.001**
No/I do not know	39.91	49%	

* proportion test, *n* = 461 respondents drinking SSB. Bold Fonts: Statistically significant difference.
